# Effect of exercise across the head and neck cancer continuum: a systematic review of randomized controlled trials

**DOI:** 10.1007/s00520-023-08126-2

**Published:** 2023-11-04

**Authors:** Alice Avancini, Anita Borsati, Lorenzo Belluomini, Diana Giannarelli, Riccardo Nocini, Jessica Insolda, Marco Sposito, Federico Schena, Michele Milella, Sara Pilotto

**Affiliations:** 1 Section of Innovation Biomedicine - Oncology Area, Department of Engineering for Innovation Medicine (DIMI), University of Verona, Verona, Italy; 2 Department of Medicine, Verona University Hospital Trust, Verona, Italy; 3 Bio-statistical Unit, Regina Elena National Cancer Institute IRCCS, Rome, Italy; 4 Section of Ears, Nose and Throat (ENT), Department of Surgical Sciences, Dentistry, Gynecology and Pediatrics, University of Verona, Verona, Italy; 5 Department of Neurosciences, Biomedicine and Movement Sciences, University of Verona, Verona, Italy

**Keywords:** Physical exercise, Physical activity, Head and neck cancer, Symptoms controls, Exercise

## Abstract

**Purpose:**

This study aims to systematically explore the impact of physical exercise as supportive therapy for head and neck cancer.

**Methods:**

A systematic search on PubMed/MEDLINE, Cochrane, and SPORTDiscus was conducted. Randomized controlled trials exploring the effects of a physical exercise intervention in comparison with usual care on outcomes in patients with head and neck cancer were selected. The RoB 2 tool was used to determine the study quality. The extracted data are reported as qualitative synthesis.

**Results:**

Among the 527 records examined, nine studies were included. No trials investigating exercise as prehabilitation were found, whereas eight studies involving 452 patients with head and neck cancer were conducted during anticancer treatment. Most trials did not report improvements in body mass index or body composition, while 2/4 and 3/5 investigations found a significant increase in muscle strength and cardiorespiratory fitness, respectively. Regarding the patients’ reported outcomes, 4 out of 7 studies observed enhancements in some domains of quality of life, and two trials out of 3 detected an amelioration in fatigue following the exercise intervention. Analyzing the exercise programs, it seems that combining aerobic and resistance training could be more beneficial compared to a single type of full-body exercise in counteracting physical decline and controlling symptoms in the anticancer therapy phase. One trial has investigated the effect of resistance exercise on patients who had terminated the anticancer treatments, reporting significant improvements in lean mass, muscle strength, and quality of life.

**Conclusion:**

Exercise may be a promising approach in patients with head and neck cancer. Future studies are needed to consolidate these results.

**Supplementary information:**

The online version contains supplementary material available at 10.1007/s00520-023-08126-2.

## Introduction

Exposure to tobacco smoking, alcohol abuse, and oncogenic viruses, including human papillomavirus and Epstein-Barr virus, are recognized risk factors that may lead to developing head and neck cancers [1]. Head and neck malignancies enclose a wide range of cancers that develop in the larynx, pharynx, oral cavity, nasal cavity, and paranasal cavity. With about 931,931 new cases and 467,125 related deaths, head and neck cancer accounts for 4.8% of all tumors and approximately 4.6% of all cancer-related deaths worldwide [2]. To date, treating head and neck cancer requires multidisciplinary expertise, encompassing surgery, radiotherapy, and systemic therapy, in order to offer the best therapeutical approach [1]. Nevertheless, during cancer care, patients with head and neck cancer may experience a series of side effects, such as fatigue, dysphagia, muscle wasting, weight loss, and functional impairments that may seriously affect patients’ quality of life [3, 4]. Additionally, changes in functional parameters and body composition after diagnosis may negatively impact patients’ disease course. For instance, in the surgical context, an observational study including 187 patients undergoing head and neck surgery found that better preoperative cardiorespiratory fitness, an objective measure to assess the individual’s functional capacity, was significantly associated with a decrease in cardiopulmonary complications [5]. Similarly, pre-surgery sarcopenia, i.e., loss of muscle mass, is a strong negative predictor of 2-year and 5-year overall survival in patients with head and neck cancer [6]. Low skeletal muscle mass has been associated with adverse outcomes, also during anticancer treatments. In this sense, sarcopenia represents a negative prognostic factor for overall survival in patients affected by head and neck cancer undergoing radiotherapy or chemoradiotherapy, and it has been significantly correlated with reduced disease-free survival, prolonged radiotherapy breaks, and chemotherapy-related toxicities [7, 8]. Moreover, such muscle impairments may persist for years after therapy conclusion, consequently altering the patients’ strength, functional capacity, and quality of life [9].

In this sense, strategies directed to improve these parameters are fundamental. Nutritional counseling has been suggested as a cornerstone in the management of head and neck cancer. Nevertheless, also exercise may be a useful strategy. Physical exercise has been established as adjunctive therapy across the cancer continuum in several cancer types, able to positively support patients by improving their physical and psychological parameters and accelerating their recovery. Exercise prehabilitation, i.e., the exercise intervention occurring between diagnosis and the start of acute treatments (and often surgery), has been shown to improve strength and cardiorespiratory fitness, reduce postoperative complications, length of hospital stay, and limit the loss of functional impairments in the postoperative phase, in different surgical cancer settings [10, 11]. During anticancer treatments, exercise interventions may improve the health-related components of host physiology, such as cardiorespiratory fitness, strength, and muscle mass [12, 13]. Moreover, exercise has been demonstrated to ameliorate several side effects of anticancer therapies, including fatigue, anemia, lymphoedema, peripheral neuropathy, and sleep quality, and enhance psychological well-being and quality of life [12, 14, 15]. In the survivorship phase, exercise may help recover from residual physical and psychological impairments, as well as preliminary data suggests that being physically active may reduce the recurrence risk [16]. Although several studies and international guidelines support the importance of exercise in cancer, most of the available review and evidence derives from lung, breast, prostate, and colorectal cancer studies. In head and neck cancer, a significant amount of literature and reviews have deeply analyzed the role of specific interventions for trismus, dysphagia, or shoulder dysfunction [17–19], whereas little is known about the impact of full-body physical exercise, defined as sessions of muscle strength and/or aerobic exercise. Therefore, this systematic review aims to investigate the impact of full-body physical exercise on physical fitness and patients’ reported outcomes, in patients with head and neck cancer, in the presurgical setting, during anticancer treatments, and after therapy conclusions in order to explore the current data and identify potential knowledge gaps to inform future studies.

## Method

### Search strategy

On October 13th, 2022, a systematic search was executed through the screening of the following electronic databases: Cochrane Central Register of Controlled Trials (CENTRAL), PubMed/MEDLINE (National Library of Medicine), and EBSCO Sports Medicine Database (SPORTDiscus). Research headings and keys included those related to head and neck anatomical district (e.g., head and neck, hypopharynx, larynx, oropharynx, oral cavity, or nasopharynx), exercise (e.g., physical activity, physical exercise, exercise prehabilitation, or prehabilitation) and cancer (e.g., tumor or malignancy), were combined using the Boolean operator “AND”. A hand-searched screen was also performed, reviewing the references list of the eligible articles and other narrative and systematic reviews. Further information regarding the literature search is available in the Supplementary Information. The present systematic review was registered on PROSPERO (CRD42022379687) and was conducted and reported according to the Preferred Reporting Items for Systematic Reviews and Meta-Analysis (PRISMA) statement [20].

### Study eligibility criteria

Following the PICO tool [21], studies were considered eligible if: i) applied a randomized controlled trial design, ii) included adult patients (age ≥ 18 years) with a confirmed diagnosis of head and neck cancer, iii) investigated a full-body exercise as intervention and utilized as a comparator, a non-exercise intervention (e.g., usual care), or placebo intervention with a minimal likelihood of muscular adaptations (e.g., Qi Qigong, stretching or relaxation), and iv) reported an outcome assessment (physical, psychological, or clinical). Regarding the intervention, exercise, according to Caspersen, was considered a planned, structured, and repetitive body movement to improve or maintain one or more components of physical fitness [22]. Additionally, the exercise intervention had to last at least 3 weeks, i.e., it must be chronic, and each repeated session must have a length of at least 10 min. Exercise interventions were categorized as aerobic, which included any activity, rhythmic in nature, that uses large muscle groups and can be performed continuously, as resistance, i.e., a form of exercise specifically designed to improve muscular strength and mass, or as combined, i.e., including both aerobic plus resistance activities. Exclusion criteria of the studies were: i) non-English full text, ii) applied other forms of physical activity or exercise as a comparator, and iii) interventions targeting specific organ sites or rehabilitation needs (e.g., exercises for tongue, swallow, or shoulder range of motion).

### Study selection and data extraction

Studies eligibility was verified through a two-step process. Initially, irrelevant references were removed after screening the title and abstract by two independent authors (A.A. and A.B.), and uncertain studies were carried on for full-text evaluation. The second step consisted of the full-text eligibility evaluation of the selected studies by the same two authors. Any discrepancies during the literature selection were resolved by discussion. For each study, the following relevant data were extracted: i) study details and characteristics: first author name, year of publication, journal of publication, country, study design; primary outcome, and secondary outcomes; ii) population: cancer sites, cancer stage, number of patients affected by human papillomavirus infection, phase of cancer continuum (i.e., prehabilitation, during anticancer treatment or after anticancer treatments), percentage of patients undergoing active anticancer treatment, number of patients allocated in the treatment group, number of patients allocated as controls; iii) exercise intervention characteristics: length of the intervention, supervision of the intervention, type of intervention, frequency, duration, intensity, progression, presence of nutritional support/intervention, presence of other interventions, type of controls; and iv) outcomes: recruitment rate, adherence rate (i.e., the percentage of training sessions successfully completed to the planned), dropouts (i.e., the ratio of patients did not complete post-intervention assessments), safety profile (i.e., the occurred adverse events of any grade), summary of the study results. To extract and collect data, a Microsoft Excel spreadsheet summary was created.

### Risk of bias assessment

The methodological quality of each included study was determined, by two independent authors (A.A. and A.B), utilizing the Cochrane Collaboration’s Risk of Bias tool 2nd version (RoB 2) [23]. The Rob2, specifically used for randomized controlled trials, assesses the following domains: i) bias arising from the randomization process; ii) bias due to deviations from intended interventions; iii) bias due to missing outcome data; iv) bias in the measurement of the outcome; and v) bias in the selection of the reported result. The summary of the risk of bias is categorized as "low risk of bias", "some concerns", or "high risk of bias" [23].

### Data synthesis

Given the scarcity of the amount of the literature and the considerable heterogeneity across studies in outcomes definition and exercise prescription, a meta-analysis was not conducted. A qualitative synthesis, in narrative form, is provided, focusing on the different timing of the cancer continuum, i.e., prior surgery (e.g., prehabilitation), during anticancer treatments, and after anticancer treatments. The extracted results were examined using tables and a narrative description, grouping data according to characteristics, as suggested by the Guidance on the Conduct of Narrative Synthesis in Systematic Reviews [24, 25].

## Results

**Fig. 1:**
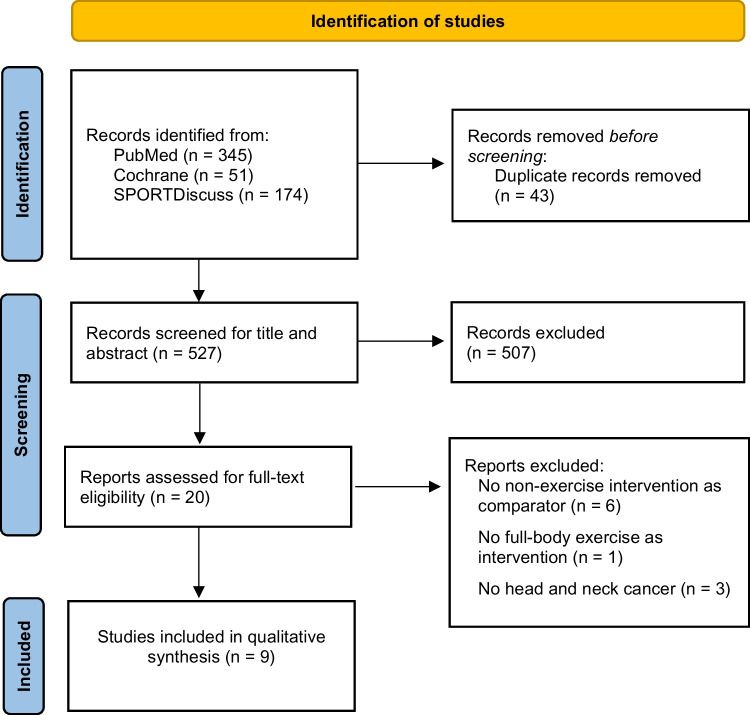
Study selection flow-chart

Among the 527 records examined for title and abstract, 506 were excluded (Fig. 1). A total of 21 research articles were screened for full-text evaluation, and among these, nine met the inclusion criteria. The reasons for the full-text exclusion are presented in the Supplementary Information.

### Characteristics of the included studies and quality assessment

The general characteristics of the included studies are presented in Table 1. Briefly, two trials were conducted in the US [26, 27], two in India [28, 29], two in Taiwan [30, 31], and one each in Canada [32], Germany [33] and Denmark [34]. Overall, seven trials applied a randomized controlled trial design [26–31] [33], whereas two performed a cross-over randomized controlled trial [32] [34]. The sample size across the studies varied from 15 to 148 patients. Six research articles specified the cancer sites across the head and neck district [26, 27, 29, 31, 32, 34], and the disease stage varied from stage I to IV, with two trials that did not report it [28, 30]. Overall, the risk of bias assessment revealed *some concerns* for eight trials, whereas, in one investigation, the risk of bias was high (Supplementary Information).

**Table 1:** Study characteristics

Author (year)	Country	Study design	Sample size (allocation)	Cancer site	Cancer stage	Anticancer treatment
*During anticancer treatment*						
Rogers L.Q. et al. (2013)	USA	RCT	15 pts (IG:7; CG: 8)	Nasopharyngeal/salivary glands/oral cavity/tongue/oropharyngeal/larynx/unknown	I–IV	Radiation or chemoradiotherapy
Samuel S.R. et al. (2013)	India	RCT	48 pts (IG: 24; CG: 24)	head and neck	NR	Chemoradiotherapy
Capozzi L.C. et al. (2016)	Canada	RCT Cross-over	60 pts (IG: 31; CG: 29)	Oral/larynx/hypopharynx/nasopharynx/oropharynx/major salivary glands/nasal cavity or paranasal sinuses/unknown	I–IV	Radiation or chemoradiotherapy
Zhao S.G. et al. (2016)	USA	RCT	20 pts (IG: 11; CG:9)	Larynx/nasopharynx/oropharynx/unknown	III–IV	Chemoradiotherapy
Grote M. et al. (2018)	Germany	RCT	20 pts (IG: 10; CG: 10)	Head and neck	I–IV	Radiation or chemoradiotherapy
Samuel S.R. et al. (2018)	India	RCT	148 pts (IG: 74; CG: 74)	Oropharynx/larynx	III–IV	Chemoradiotherapy
Yen C.J. et al. (2019)	Taiwan	RCT	84 pts (IG: 42; CG: 42)	Head and neck	NR	Chemotherapy
Lin K.Y. et al. (2021)	Taiwan	RCT	57 pts (IG: 29; CG: 28)	Tongue/nasopharyngeal/oropharyngeal parotid	I–IV	Chemotherapy
*After anticancer treatment*						
Lønbro S. et al. (2013)	Denmark	RCT cross-over	41 pts (IG: 20; CG: 21)	Pharynx/larynx/oral cavity/unknown	I–IV	Radiation or chemoradiotherapy

*RCT* randomized controlled trial, *IG* interventional group, *CG* control group *NR* not reported

### Exercise before surgery

No randomized controlled trials have currently explored the effect of exercise as prehabilitation in patients with head and neck cancer.

### Exercise during oncological treatments

Eight trials have investigated the effect of exercise in patients with head and neck cancer during active anticancer treatments [26–33]. A total of three studies were conducted on patients treated with radiation or chemoradiotherapy [26, 32, 33], three in patients scheduled for chemoradiotherapy [27–29], and two in those undergoing chemotherapy [30, 31].

Regardless of exercise prescription (Table 2.), the length of intervention ranged from 7 to 12 weeks. Five out of eight studies proposed a combined exercise, i.e., aerobic plus resistance training [27–31], whereas three focused on resistance training only [26, 32, 33]. The exercise frequency varied 2–5 days a week, and each session lasted approximately 30–90 min. Two studies did not report the exercise intensity [26, 32, 33], whereas four investigations declared to have performed a progressive exercise prescription [26, 31–33]. Concerning supervision, three studies stated to start the exercise intervention with a supervised phase followed by an unsupervised period [26, 27, 29], three proposed a supervised program [28, 31, 33], and one a mixed program with supervised and unsupervised sessions in the same week [32].

**Table 2:** Description of the exercise intervention programs of the studies included in the systematic review

Author	Exercise intervention	Progression	Recruitment / adherence rate	Safety	Dropouts
*During anticancer treatment*					
Rogers L.Q. et al. (2013)	6 weeks supervised + 6 weeks unsupervised resistance training, twice a week, 9 exercises vs*.* usual care	Yes	RR: 45.0% AR: 83% (supervised); 62% (unsupervised)	CG: non-EX related left arm pain (non-serious); hospital admission for narcotic-related dehydration and constipation (serious); IG: non-EX related hospital admission for infectious diarrhea	IG:28,6%; CG:0%
Samuel S.R. et al. (2013)	11 weeks of supervised aerobic training 15/20 min per session at 3–5 RPE + resistance training, 2–3 sets of 8–15 reps at 3–5 RPE, 5 days per week vs. usual care	NR	RR: 92.3% AR:NR	No	IG:16,7%; CG: 4,2%
Capozzi L.C. et al. (2016)	12 weeks of supervised and unsupervised resistance training four times per week (twice supervised + twice home-based), 10 exercises, 2–3 sets for 8 reps vs. usual care	Yes	RR: 55.6% AR: 45.2%	NR	IG:38.7%; CG:20.7%
Zhao S.G. et al. (2016)	7 weeks of supervised + 7 weeks of unsupervised walking training + resistance training of 3 sets of 8–12 reps at 11–13 RPE, thrice a week for the supervised period, five times per week during the unsupervised period vs. usual care	NR	RR: NR AR: 72.0%	NR	IG:0.0%; CG:22.2%
Grote M. et al. (2018)	7 weeks of supervised resistance training thrice a week, 3 exercises, 3 sets of 8–12 reps vs. usual care	Yes	RR: 20.0% AR: 90.0%	non-EX related pain (for mucositis)/ abdominal tension (for endoscopic gastrostomy)	NR
Samuel S.R. et al. (2018)	7 weeks of supervised + 6 weeks unsupervised aerobic training 15/20 min per session at 3–5 RPE + resistance training, 1 set of 8–15 reps at 3–5 RPE, 5 days per week vs. usual care	NR	RR:60.2% AR:NR	NR	IG: 21,6%; CG: 10.8%
Yen C.J. et al. (2019)	8 weeks of aerobic training, 30 min per session at 60/70% HRmax + resistance training, 10 exercises, 2 sets of 10 repetition at 3–5 RPE vs. usual care	NR	RR: NR AR: NR	NR	IG: 19.1%; CG: 9.5%
Lin K.Y. et al. (2021)	8 weeks of supervised aerobic training, 30 min per session at 60/80% of HRmax + moderate/hard resistance training, 1–3 sets of 8–12 reps, thrice a week vs*.* usual care	Yes	RR: 60.6% AR: 93.1%	NR	IG: 31%; CG:28,6%
*After anticancer treatment*					
Lønbro S. et al. (2013)	12 weeks of supervised and unsupervised resistance training, 2.5 times per week, 7 exercises, 2–3sets of 8–15 reps vs*.* waiting list	NR	RR:22% AR: 91%	No adverse events were registered	IG: 5%; CG: 19%

*NR* not reported, *RR* recruitment rate, *AR* adherence rate, *IG* interventional group, *CG* control group, *BMI* body mass index, *6MWT* 6-min walking test, *QoL* quality of life. The arrow up (↑) symbol denotes significant improvement; the arrow down (↓) symbol denotes significant worsening; the double head arrow ν(↔) denotes no significant change

#### Safety and feasibility

Feasibility was the primary aim in three trials [26, 27, 33], assessed through recruitment rate, adherence rate to the exercise intervention, and withdrawals. Six out of eight trials reported a recruitment rate ranging from 20 to 32.3% [26, 28, 29, 32, 33, 35]. Adherence to the exercise intervention was provided by five studies and varied from 45.2% to 93.1% [26, 27, 31–33]. Only one investigation did not report information regarding withdrawal [33]. Dropout rates among the patients allocated in the intervention group ranged from 0 to 38.7%, whereas for controls varied between 0 and 28.6% [26–32]. Regarding safety, only three studies reported the safety profile of exercise [26, 28, 33]. Samuel and colleagues stated that no adverse events occurred during the study [28], whereas in the investigations of Grote et al. [33] and Rogers et al. [26], non-exercise-related adverse events were reported **(**Table 2**).**

#### Anthropometric measures and body composition

Five studies have analyzed the effect of an exercise intervention on body mass index (BMI) (Table 3.). One trial reported a significant decline in BMI in the intervention groups compared to the controls [30], while one investigation found a maintenance of the BMI [31]. Three studies reported a decline in BMI in the intervention groups, which was not significantly different from this experienced by the controls [26, 27, 32].

**Table 3:** Main results of the studies included in the systematic review

Study	Main results
*During anticancer treatment*	
Rogers L.Q. et al. (2013)	• No differences for lean body mass (decrease in both groups); IG: 131.1 ± 26.5 kg vs. 113.8 ± 32.9 kg; CG: 138.5 ± 39.1 kg vs. 132.4 ± 32.8 kg (*p* > 0.05; *d* = − 0.40) • No significant changes for body mass index (decrease more in IG); IG: 28.4 ± 9.0 kg/m^2^ vs. 26.4 ± 9.4 kg/m^2^; CG: 31.3 ± 8.0 kg/m^2^ vs. 29.9 ± 7.2 kg/m^2^ (*p* > 0.05; *d* = − 0.29) • No changes in knee extensors strength (increase in IG); IG: 107.9 ± 37.8 kg vs. 115.0 ± 54.4 kg; CG: 94.2 ± 46.4 kg vs. 92.1 ± 41.3 kg (*p* > 0.05; *d* = − 0.19) • Improvements in emotional well-being; IG: 16 ± 5.5 points vs. 19.4 ± 5.5 points; CG: 18.7 ± 3.7 points vs. 21.6 ± 2.4 points (*p* < 0.05; *d* = − 0.45) • No significant differences in fatigue levels; IG: 14.4 ± 6.7 points vs. 19.0 ± 10.0 points; CG: 10.1 ± 7.4 points vs. 16.5 ± 11.1 points (p > 0.05; d = − 0.27) • No significant differences in physical functioning; IG: 10.0 ± 1.2 points vs. 10.2 ± 1.3 points; CG: 9.0 ± 2.7 points vs. 8.8 ± 2.0 points (p < 0.05; d = 0.19) • No differences in physical well-being (decrease in both groups); IG: 20.8 ± 4.8 points vs. 19.8 ± 7.0 points; CG: 23.9 ± 4.0 points vs. 20.6 ± 6.3 points. (*p* > 0.05) • No differences in social well-being (decrease in both groups); IG: 21.2 ± 5.6 points vs. 18.2 ± 8.6 points; CG: 26.4 ± 1.7 points vs. 24.8 ± 3.9 points. (*p* > 0.05 *d* = 0.36) • No differences in functional well-being (decrease in both groups); IG: 15.7 ± 43. points vs. 13.2 ± 4.7 points; CG: 21.2 ± 5.3 points vs. 17.6 ± 5.4 points. (*p* > 0.05; *d* = 0.33) • No significant differences in total FACT-H&N domain (decrease in both groups); IG: 110.8 ± 16.0 points vs. 103.0 ± 26.7 points; CG: 125.9 ± 15.0 points vs. 118.4 ± 16.6 points (*p* > 0.05; *d* = 0.03) and in the FACT-G (decrease in both groups); IG: 73.8 ± 14.8 points vs. 170.6 ± 18.2 points; CG: 90.4 ± 10.8 points vs. 84.6 ± 13.8 points (*p* > 0.05; *d* = 0.39)
Samuel S.R. et al. (2013)	• A significant increase in functional capacity in IG and a significant decrease in CG; IG: 408 m; CI: 390 to 465 vs. 450 m CI: 417.2 to 495.6 (*p* = 0.039); CG:396 m CI: 335.1 to 441 vs. 300 CI: 201 to 390 (*p* < 0.001) • No changes for physical component score (maintenance for IG, deterioration for CG); IG: 43.2 points; CI:39.57 to 45.07 vs. 43.0 points; CI: 38.47 to 48.4 (*p* = 0.48); CG: 38.6 points; CI:30.72 to 44.6 vs 32.7 points; CI:28.3 to 38.6 (*p* = 0.06)
Capozzi L.C. et al. (2016)	• No differences in lean body mass between groups (decline in both groups); IG: − 4.9 ± 0.7 kg vs. − 5.4 ± 0.7 kg (mean difference from baseline to post-intervention) (*p* = 0.73, *d* = 0.13) • No differences in the percentage of body fat between groups (decline in both groups); IG: − 1.5 ± 0.5% vs. − 1.9 ± 0.5% (mean difference from baseline to post-intervention) (*p* = 0.66, *d* = 0.15) • No differences in BMI between groups (decline in both groups); IG: − 2.5 ± 0.3 kg/m^2^ vs. − 2.8 ± 0.3 kg/m^2^ (mean difference from baseline to post-intervention) (*p* = 0.39; *d* = 0.18) • No differences in functional capacity between groups (decline in both groups); IG: − 13 ± 19.6 m vs. − 35.4 ± 18.7 m (mean difference from baseline to post-intervention) (*p* = 0.59; *d* = 0.21) • No differences in handgrip strength between groups (decline in both groups); IG: − 3.0 ± 1.4 kg vs. − 6.7 ± 1.2 kg (mean difference from baseline to post-intervention) (*p* = 0.73; *d* = 0.52) • No differences in flexibility between groups (increase in both groups); IG: 2.7 ± 0.9 cm vs. 0.8 ± 0.8 cm (mean difference from baseline to post-intervention) (*p* = 0.66; *d* = 0.41) • No differences in the total amount of physical activity between groups; IG: 71.7 ± 76.0 min vs − 61.6 ± 71.3 min (mean difference from baseline to post-intervention) (*p* = 0.06; *d* = 0.54) • No differences in quality of life score between groups (decline in both groups); IG: − 21.3 ± 4.4 points vs. − 19.1 ± 4.1 points (mean difference from baseline to post-intervention) (*p* = 0.75; *d* = − 0.09) • No differences in depression between groups (increase in both groups); IG: 5.1 ± 1.9 points vs. 4.9 ± 1.7 points (mean difference from baseline to post-intervention) (*p* = 0.87; − 0.23) • No differences in nutritional status between groups (increase in both groups, i.e. malnutrition); IG: 4.4 ± 1.5 points vs 6.7 ± 1.4 points (mean difference from baseline to post-intervention) (*p* = − 0.37; *d* = − 0.29)
Zhao S.G. et al. (2016)	• No significant difference in lean body mass (improvements in both groups); IG: 4.7 ± 1.5 kg vs 4.0 ± 0.9 kg (mean difference from baseline to post-intervention) • No significant differences in BMI (decline in both groups); IG: − 3,9 ± 0.7 vs − 4.6 ± 0.6 kg/m^2^ (mean difference from baseline to post-intervention) • No significant changes in functional capacity (increase in IG, decrease in CG); IG: 60 ± 40 vs − 19 ± 89 (feet) (mean difference from baseline to post-intervention) • No significant changes in elbow flexion isokinetic dynamometer (mean difference from baseline to post-intervention) • No differences in balance (in both groups); IG: − 07 ± 0.6 s vs. − 0.2 ± 0.6 s (mean difference from baseline to post-intervention) • No changes in physical activity levels; IG: 0.0 ± 0.01 min vs. − 0.03 ± 0.01 min (mean difference from baseline to post-intervention) • No significant differences in physical summary; IG: 1 ± 4 points vs. − 3 ± 8 points (mean difference from baseline to post-intervention) • No significant differences in mental summary; IG: 9 ± 4 points vs. − 9 ± 12 points (mean difference from baseline to post-intervention) • No significant changes in the vitality subscale; IG: 7 ± 5 points vs. − 9 ± 10 points (mean difference from baseline to post-intervention) • Improvements in mental health subscale for IG and decrease in CG; IG: 15 ± 4 points vs. − 1 ± 6 points (mean difference from baseline to post-intervention) • No changes in sleep quality (decline in both groups); IG: − 14 ± 5 points vs. − 1 ± 11 points (mean difference from baseline to post-intervention) • No significant differences in fruit and vegetable servings, solid food servings, and meal replacements between groups • No differences in concurrent chemoradiotherapy toxicities between groups
Grote M. et al. (2018)	• No differences for lean body mass (decease in both groups): IG: 58.6 ± 4.9 kg vs. 59.1 ± 7.1 kg; CG: 54.4 ± 12.5 kg vs. 52.7 ± 12.1 kg (*p* = 0.27) • No differences for fat mass (decrease in both groups): IG: 14.7 ± 8.6 kg vs. 10.2 ± 6.6 kg; CG 16.4 ± 7.0 kg vs. 31.2 ± 5.3 kg (*p* = 0.55) • No differences for quality of life (decrease in both groups): IG:80.1 ± 11.2 points vs. 64.4 ± 18.4 points; CG:75.7 ± 18.8 points vs. 59.5 ± 26.5 points (*p* = 0.89) • No differences for general fatigue: IG:11.3 ± 3.7 points vs. 11.8 ± 4.3 points; CG:10.1 ± 2.9 points vs. 11.9 ± 4.6 points (*p* = 0.87) • No differences for physical fatigue: IG:12.0 ± 5.0 points vs. 13.3 ± 5.0 points; CG:10.0 ± 2.9 points vs. 11.8 ± 5.1 points (*p* = 0.48) • No differences for mental fatigue: IG:7.6 ± 4.9 points vs. 8.3 ± 2.3 points; CG:8.3 ± 3.4 points vs. 9.2 ± 2.3 points (*p* = 0.69) • No differences for reduced activity: IG:11.5 ± 5.0 points vs. 12.4 ± 4.4 points; CG:11.4 ± 2.7 points vs. 12.6 ± 4.2 points (*p* = 0.70) • No differences for motivation: IG:8.6 ± 4.2 points vs. 8.0 ± 2.5 points; CG:9.0 ± 3.99 points vs. 10.3 ± 4.8 points (*p* = 0.44)
Samuel S.R. et al. (2019)	• Significant increase in the exercise group and decrease in the controls for functional capacity: IG: 446.31 ± 62.87 m vs. 483.16 ± 88.24 m; CG: 447.32 ± 59.22 m vs. 374.52 ± 110.26 m (*p* < 0.001) • No differences for hemoglobin and platelets values; decrease of 14% in the hemoglobin value for both groups (*p* = 0.61); decrease of 8.68% for controls and 12.19% for exercise groups in the platelets value (*p* = 0.57) • Significant increase in quality of life for physical component: IG: 43.96 ± 7.21 points vs. 48.58 ± 6.63 points; CG: 43.51 ± 7.10 points vs. 39.10 ± 4.95 points (*p* < 0.001) • Significant increase in quality of life for mental component: IG: 39.58 ± 9.85 points vs. 40.78 ± 7.79 points; CG: 42.64 ± 7.47 points vs. 36.34 ± 5.20 points (*p* < 0.001) • Significant improvement in the fatigue level: IG: 3.70 ± 1.75 points vs. 2.45 ± 1.97 points; CG: 2.91 ± 1.84 points vs. 4.48 ± 1.59 points (*p* < 0.001)
Yen C.J et al. (2019)	• Significant increase in the skeletal muscle rate: IG: 32.1 ± 3.8 vs. 33.6 ± 4.1 (*p* < 0,05); CG: 31.8 ± 4.6 vs. 32.3 ± 4.0 (*p* > 0.05) • Significant decrease in the visceral fat: IG:7.9 ± 4.7 vs. 7.4 ± 4.5 (*p* < 0,05); CG: 8.2 ± 5.5 vs. 7.8 ± 5.7 (*p* > 0.05) • No differences for total fat mass: IG: 23.6 ± 6.3% vs 23.1 ± 5.3% (*p* > 0.05); CG: 24.1 ± 6.3% vs 23.6 ± 7.9% (*p* > 0.05) • Significant decrease in the body mass index for the controls: IG: 22.4 ± 2.9 kg/m^2^ vs. 22.1 ± 2.9 kg/m^2^ (*p* > 0.05); CG: 22.3 ± 3.8 kg/m^2^ vs. 21.9 ± 4.0 kg/m^2^ (*p* < 0.05) • Significant increase in the exercise group and decrease in the controls for functional capacity: IG: 410.1 ± 74.7 m vs. 543.8 ± 54.0 m (*p* < 0.05); CG: 392.2 ± 119.2 m vs. 353.4 ± 84.9 m (*p* < 0.05) • Significant decrease in resting systolic blood pressure: IG: 114,4 ± 18.7 mmHg vs. 104.2 ± 11.1 mmHg; CG: 120.7 ± 18.3 mmHg vs 116.0 ± 15.6 mmHg (*p* < 0.05) • Significant decrease in resting diastolic blood pressure: IG: 70.9 ± 13.3 mmHg vs. 61.9 ± 8.9 mmHg; CG: 74.3 ± 12.7 mmHg vs. 75.8 ± 10.3 mmHg (*p* < 0.05) • Significant decrease in the exercise group and increase in the controls for resting heart rate: IG:83.3 ± 12.4 bpm vs. 73.5 ± 11.1 bpm; CG:77.9 ± 16.5 bpm vs. 83.2 ± 17.8 bpm (*p* < 0.05)
Lin K.Y. et al. (2021)	• Significant improvements in the skeletal muscle percentage: IG: 34.1 ± 3.4% vs. 34.5 ± 2.4% (*p* = 0.61); CG: 31.5 ± 2.9% vs. 31.4 ± 2.4% (*p* = 0.87) (between groups *p* = 0.008) • Significant improvement in the body fat percentage: IG: 25.5 ± 4.0% vs. 21.0 ± 2.8% (*p* = 0.35); CG: 25.9 ± 3.5% vs 25.8 ± 2.5% (*p* = 0.49) (between groups *p* = 0.002) • No significant difference for visceral fat level: IG: 6.9 ± 4.8 vs 6.7 ± 4.8 (*p* = 0.32); CG: 8.9 ± 5.1 vs 8.9 ± 4.9 (*p* = 0.80) • No significant difference for body mass index: IG: 21.2 ± 3.4 kg/m^2^ vs. 21.1 ± 3.3 kg/m^2^ (*p* = 0.87); CG: 23.1 ± 3.2 kg/m^2^ vs. 23.2 ± 3.3 kg/m^2^ (*p* = 0.84) (between groups *p* = 0.139) • Significant decrease in the controls for functional capacity, maintenance in the exercise groups: IG: 56.7 ± 10.1 vs 64.7 ± 25.1 (*p* = 0.24); CG: 78.0 ± 25.1 vs 67.6 ± 19.8 (*p* = 0.03) (between groups *p* = 0.503) • Significant increase in the exercise groups, non-significant decrease in the controls for upper limb strength: IG: 24.1 ± 6.3 repetitions vs. 27.0 ± 5.8 repetitions (*p* = 0.027); CG: 23.4 ± 8.4 repetitions vs. 21.06 ± 5.38 repetitions (*p* = 0.093) (between group *p* = 0.037) • Maintenance in the exercise groups and significant decrease in the controls for lower limb strength: IG: 19.7 ± 6.58 repetitions vs. 20.14 ± 7.04 repetitions (*p* = 0.752) CG: 15.6 ± 4.37 repetitions vs. 13.1 ± 3.87 repetitions (*p* = 0.013) (between groups *p* = 0.025) • No significant difference for balance: IG: 7.29 ± 2.21 s vs. 6.42 ± 1.51 s (*p* = 0.196); CG: 8.3 ± 1.29 s vs. 8.4 ± 1.29 s (*p* = 0.71) (between groups *p* = 0.01) • Significant improve in the upper limb flexibility: IG: − 19.6 ± 16.7 cm vs. − 11 ± 16.6 cm (*p* = 0.01) CG: − 9.2 ± 14.4 cm vs. − 12.3 ± 16.6 cm (*p* = 0.01) (between groups *p* = 0.83) • Significant improve in the lower limb flexibility: IG: − 0.92 ± 5.1 cm vs. 4.28 ± 4.19 cm (*p* = 0.02) CG:4.0 ± 10.8 cm vs. 0.2 ± 11.6 cm (*p* = 0.19) (between groups *p* = 0.94) • Significant increase in the exercise groups for quality-of-life domains: global health status, pain, and weight gain • Significant increase in both groups for quality-of-life domains: physical functioning, emotional functioning, social functioning, fatigue, nausea and vomiting, appetite loss, sense problems, speech problem, and feeling ill • Significant increase in the controls for quality-of-life domain: constipation • No significant difference for quality-of-life domains: dyspnea, insomnia, diarrhea, financial difficulties, oral pain, swallowing problems, social contact problems, sex problems, teeth problems, mouth opening problems, dry mouth, sticky saliva, coughing, pain killers, nutritional supplements, feeding tube, and weight loss
*After anticancer treatment*	
Lønbro S. et al. (2013)	• No significant difference from baseline to week 24 for body composition, body weight, strength, and quality of life • In the first 12-week, early exercise (EE) group increased lean body mass by 4.3% (2.3 kg; *p* < 0.001; 95% CI: 0.5 to 2.5) • In the last 24-week, delayed exercise (DE) group increased lean body mass by 4.2% (2.4 kg; *p* < 0.001; 95% CI: 1.1 to 3.1) • In the first 12-week, EE group increased isometric knee extensor strength by 20% (33 Nm; *p* < 0.001; 95% CI: 16 to 50) • In the last 24-week, DE group increased isometric knee extensor strength by 21% (34 Nm kg; *p* < 0.001; 95% CI: 17 to 50) • Similar results were observed for knee flexor strength, arm curl, chair rise, and quality of life

*IG* interventional group, *CG* control group

Body composition components were evaluated in six trials using dual-energy x-ray absorptiometry [27, 32] or bioimpedance analysis [26, 30, 31, 33]. Focusing on lean body mass, four investigations did not observe significant differences, whereas the remaining two found significant changes. Among those that did not report significative improvements, two studies exploring the effect of 12 weeks of progressive resistance training versus usual care reported a non-statistically significative decline in lean body mass in both intervention and control groups [26, 32], one detected an increase in the intervention and a slight decrease in the controls, but the differences did not result to be significative [33]; another one, which tested the combination of 14-week of supervised and unsupervised walking and resistance exercises found non-significant improvements in both groups [27]. On the other hand, two studies that proposed a combined aerobic and resistance exercise training observed significant improvement in skeletal muscle mass [30, 31]. For instance, Lin and colleagues proposed moderate-intensity aerobic training on the treadmill and resistance exercises using elastic bands or free weights to perform three times per week. After eight weeks, although the changes within groups were not significant (interventional group (IG): 34.1 ± 3.4% pre-intervention, 34.5 ± 2.4% post-intervention, *p* = 0.61 vs. control groups (CG): 31.5 ± 2.9% pre-intervention, 31.4 ± 2.4% post-intervention, *p* = 0.87), the difference between groups has reached the statistical significance (*p* = 0.008) [31].

The fat component was reported in four trials, two observed a non-significant decline in both groups [32, 33]. Instead, a study including 84 patients found that 8 weeks of aerobic and resistance training at moderate intensity was able to reduce visceral fat (IG:7.9 ± 4.7 pre-intervention, 7.4 ± 4.5 post-intervention *p* < 0,05 vs. CG: 8.2 ± 5.5 pre-intervention, 7.8 ± 5.7 post-intervention, *p*-value not reported) but did not produce significative alterations in the total body fat [30]. On the contrary, the just mentioned trial by Lin et al., which proposed a similar exercise intervention in 57 patients, found a decrease in the total body fat, which resulted in a significant difference between groups (IG: 25.5 ± 4.0 pre-intervention, 21.0 ± 2.8 post-intervention vs*.* CG: 25.9 ± 3.5 pre- intervention, 25.8 ± 2.5 post-intervention, *p* = 0.002), but did not observe alterations in visceral body fat [31].

#### Physical function measures

Different outcomes related to physical function were evaluated in response to exercise intervention, including cardiorespiratory fitness, muscle strength, flexibility, and balance (Table 3.).

Cardiorespiratory fitness was indirectly estimated using the “six minutes walking test” in five trials [27–30, 32] and the “three minutes step test" in one investigation [31]. A 6-week supervised resistance training followed by 6 other weeks of home-based resistance exercises did not produce improvements in cardiorespiratory fitness, but even a decline was reported in both groups, interventional and control (mean change from baseline to post-intervention: IG: − 13 ± 19.6 m vs*.* − 35.4 ± 18.7 m (*p* = 0.59) [33]. Two investigations reported an increase in cardiorespiratory fitness among the interventional groups and a decrease in the controls, but the differences were not statistically significant [27, 31]. Three trials found that a combined aerobic and resistance exercise intervention significantly improved cardiorespiratory fitness compared to usual care [28–30]. For instance, an 11-week (7 weeks of supervised followed by 4 weeks of home-based intervention) of 15–20 min of aerobic plus resistance training performed at a moderate intensity 5 days per week increased by 37 m the performance in the “Six minutes walking test”, whereas the controls experienced a decrease of 73 m, resulting in a significant difference between groups (IG: 446.31 ± 62.87 m pre-intervention, 483.16 ± 88.24 m post-intervention vs. CG: 447.32 ± 59.22 m pre-intervention, 374.52 ± 110.26 m post-intervention, *p* < 0.001) [29].

Muscular strength was assessed in four trials [26, 27, 31–33], using isokinetic dynamometers, functional tests, such as “30 s arm curl and chair stand”, and the handgrip strength test. Two studies that tested 12 weeks of resistance training did not find improvements in muscle strength [26, 32]. In the study of Capozzi et al., a 12-week resistance training performed four times per week was unable to preserve muscular strength since, in the post-intervention evaluations, a decline in both groups, intervention, and control, was observed [33]. Similarly, in the investigation of Rogers and colleagues, even if a reduction was not detected, no significant changes were reported in the strength measures [26]. Instead, two trials combining aerobic training with resistance exercises found improvements in muscle strength measures [27, 31]. In the study of Lin et al., after exercise intervention, a significant difference between groups in favor of exercise groups was observed for the upper limb (IG: 24.1 ± 6.3 repetitions pre-intervention, 27.0 ± 5.8 repetitions post-intervention vs. CG: 23.4 ± 8.4 repetitions pre-intervention, 21.06 ± 5.38 repetitions post-intervention, *p* = 0.037) and lower limb strength (IG: 19.7 ± 6.58 repetitions pre-intervention, 20.14 ± 7.04 repetitions post-intervention vs. CG: 15.6 ± 4.37 repetitions pre-intervention, 13.1 ± 3.87 repetitions post-intervention, *p* = 0.025) [31]. Zhao and colleagues, on the one side, did not detect differences in upper limb strength but reported a maintenance in knee extensor strength for the exercise groups and a decline in the controls, finally resulting in a significant difference between groups (mean change from baseline to post-intervention, IG: − 4 ± 7 N-m vs*.* CG: − 46 ± 14 N-m, *p* < 0.05) [27].

Balance and flexibility were assessed in two studies each. Regarding balance, any intervention has produced improvements [27, 31], whereas for flexibility, one investigation reported no significant change [32], and one found an increase in upper limb flexibility [31].

#### Other outcomes

Across the studies, blood pressure, resting heart rate, the total count of hemoglobin and platelets, and treatment toxicities were evaluated once (Table 3.). In the study of Zhao et al. [27], treatment toxicities, assessed according to the Common Toxicity Criteria for Adverse Events, did not differ between exercise and control groups. Similarly, the levels of hemoglobin and platelets did not result in a significant change between the two groups, which exhibited a decline over time [29]. In the study of Yen and colleagues, the 8-week exercise intervention produced a significant reduction in rest systolic (IG: 114.4 ± 18.7 mmHg pre-intervention, 104.2 ± 11.1 mmHg post-intervention vs*.* CG: 120.7 ± 18.3 mmHg pre-intervention, 116.0 ± 15.6 mmHg post-intervention, *p* < 0.05) and diastolic pressure (IG: 70.9 ± 13.3 mmHg pre-intervention, 61.9 ± 8.9 mmHg post-intervention vs*.* CG: 74.3 ± 12.7 mmHg pre-intervention, 75.8 ± 10.3 mmHg post-intervention, *p* < 0.05), as well as in resting heart rate (IG: 83.3 ± 12.4 bpm pre-intervention, 73.5 ± 11.1 bpm post-intervention vs. CG: 77.9 ± 16.5 bpm pre-intervention, 83.2 ± 17.8 bpm post-intervention, *p* < 0.05) [30].

#### Patient-reported outcomes

The quality of life was examined in seven out of eight trials (Table 3.). Different scales were used, including Functional Assessment of Cancer Therapy (FACT)-Anemia [32], Functional Assessment of Cancer Therapy Head and Neck Cancer Symptom Index (FHNSI)—22 [32], Short Form Health Survey 36 (SF-36) [27–29], Functional Assessment of Anorexia/cachexia Therapy (FAACT) [33], European Organization for Research and Treatment of Cancer (EORTC) QLQ-C30 [31], FACT—Head and Neck [26], FACT—general [26], EORTC QLQ Head and Neck 35 [31]. Two studies that proposed resistance training as an intervention did not report enhancements in quality of life domains [32, 33]. Three investigations exploring combined aerobic and resistance training reported an improvement in the mental health variable measured with the SF-36 [27–29], while one of these also found an increase in the physical component [29]. Rogers described an increase in emotional well-being and no significant change in fatigue, physical functioning, social well-being, functional well-being, and the total score [26]. In the study of Lin and colleagues, the exercise group exhibited a better profile in the global health status, physical, role, and emotional functioning, appetite loss, and fatigue compared to controls [31]. Additionally, two studies assessed fatigue using specific tools. Grote and colleagues did not find improvement in the fatigue variables using the Multidimensional Fatigue Inventory [33], whereas a significant decrease was reported in the study of Samuel et al., which evaluated it with the National Comprehensive Cancer Network numeric rating scale 0–10 (IG: 3.70 ± 1.75 point pre-intervention, 2.45 ± 1.97 point post-intervention vs*.* CG: 2.91 ± 1.84 point pre-intervention, 4.48 ± 1.59 point post-intervention, *p* < 0.001) [31]. Depression, nutritional status, and sleep were assessed in one study each; no significant improvements were detected [27, 32].

### Exercise after oncological treatments

One trial included patients affected by head and neck cancer after the oncologic treatment phase [34] (Table 3.). In this cross-over randomized controlled trial, patients were randomized to participate in an early exercise intervention (EAI) composed of 12 weeks of progressive resistance training or usual care (self-chosen physical activity). Subsequently, the groups reversed the interventions, i.e., patients enrolled in the self-chosen physical activity underwent resistance training (delayed exercise intervention—DEI), and the EAI group acted as a control. The study reported a recruitment rate of 22%, an overall adherence to resistance training of 95%, and no adverse events were registered. After the first 12 weeks, the EAI group increased by 4.3% lean body mass, while the DEI patients had a change of 1.3% (*p* = 0.0005). After the last 12 weeks, the DEI groups experienced a gain of 4.2% in lean body mass, while in the EAI only a 0.5% improvement was detected (*p* < 0.0001). Similar results were obtained for muscle strength, evaluated with isometric and isokinetic knee extensors and flexors, and aerobic capacity measured with the stair climb test, which significantly increased in EAI compared to DEI after the first 12 weeks, while after the last 12 weeks showed a reverse trend, i.e., significantly improve in the DEI than EAI group. Regarding the quality of life, resistance training intervention enhanced the global health status and cognitive function in the EAI, and improved physical function in the DEI group. Considering the entire study period, i.e., 24 weeks, both interventions exhibited improvements in lean body mass, strength, aerobic capacity, and quality of life without differences between groups [34].

## Discussion

This systematic review provides a comprehensive overview of trials investigating the role of full-body physical exercise after a diagnosis of head and neck cancer. A total of nine randomized controlled trials were identified, conducted during oncological treatment (*n* = 8), after the conclusion of anticancer therapies (*n* = 1), whereas none was performed before treatments, i.e., as prehabilitation.

The last update of the Enhanced Recovery After Surgery (ERAS) guidelines highlighted the overall aim to improve early functional recovery in patients undergoing major head and neck cancer surgery [36]. Among the proposed multimodal interventions, prehabilitation, aiming to boost the patient’s condition before surgery, plays a key role. In the last ERAS guidelines for head and neck cancer surgery, prehabilitation has primarily focused on optimizing nutritional status and intervention to reverse malnutrition or diminish its risk, whereas no mention was made of exercise [36]. It is not surprising, given the absence of evidence in this setting. However, preoperative exercise might be an important intervention to increase muscle mass and cardiorespiratory fitness, which are prognostic factors before head and neck cancer surgery [5, 6]. Moreover, from experiences in other surgical contexts, including thoracic or abdominal surgery, preoperative exercise may reduce postoperative complications and length of hospital stay and diminish the loss of function [10, 11]. It is possible to speculate that these benefits may also be achieved in people affected by head and neck cancers. Three clinical trials exploring the impact of exercise as prehabilitation are currently ongoing to address these issues (Table 4.). These investigations will probably pave the way for exercise prehabilitation in the head and neck cancer setting, provide important preliminary information, and extend the base of evidence about the potential contribution of exercise in this setting.

**Table 4:** Ongoing studies on physical exercise in head and neck cancer setting

Number identifier	Country	Title	Timing	Sample size/study design	Intervention	Primary outcome
NCT05256238	Belgium	Improving health-related quality of life of head and neck cancer patients via a dedicated comprehensive supervised exercise program (CSEP)	During + after anticancer treatment	150 pts RCT	Supervised and unsupervised exercise sessions vs. usual supportive care	QoL
NCT05432297	Sweden	Preventive randomized study regarding physical activity intervention for patients receiving radiotherapy for head and neck cancer: the effect of preventive intervention for physical activity, function, and quality of life	During anticancer treatment	80 pts RCT	Personalized physical activity vs. usual care (advice)	Physical activity
NCT04788264	USA	A pilot study to assess feasibility of a clinically significant increase in physical activity in patients with head and neck cancer undergoing active treatment	During anticancer treatment	20 pts Single arm	Personalized exercise prescription using Fitbit	Daily steps
NCT04658706	Spain	Supervised exercise for head and neck cancer patients initiated previously or after treatment: the SEHNECA randomized controlled trial	Prior treatment and after treatment	120 pts Three-arm RCT	Two weeks of exercise prehabilitation vs.12 weeks of exercise after treatment conclusion vs. usual care	Lean body mass
NCT05418842	Canada	Exercise prehabilitation in patients with head and neck squamous-cell carcinoma: the FIT4TREAT trial	Prior treatment	46 pts RCT	High-intensity interval training + resistance training vs. usual care	Functional capacity
NCT04598087	Canada	Multiphasic prehabilitation in patients undergoing surgery for head and neck cancer	Prior, during, and after treatment	96 pts Single group	Tailored exercise prescription involving aerobic and resistance training	QoL

*QoL* quality of life, *RCT* randomized controlled trial, *pts* patients

Regarding exercise intervention during and after anticancer treatments, the adherence rate across the studies was highly heterogeneous, suggesting that future studies are needed to explore programs addressed to increase this outcome. The optimization of adherence to an exercise program is crucial, particularly because a head and neck cancer diagnosis profoundly impacts patients, also affecting their lifestyle. Indeed a survey by Rogers and colleagues reported that if 30.5% of patients met the exercise guidelines before the disease, such percentage drastically dropped to 8.5% after diagnosis [37]. Despite the high prevalence of insufficient exercise levels, patients with head and neck cancer are interested in exercising, with 75% willing to start an exercise program [38]. Barriers related to exercise may influence the adoption and maintenance of an active lifestyle. Patients may face a series of obstacles, such as dry mouth, difficulty eating, pain, fatigue, and shortness of breath, which are typically the direct consequences of head and neck anticancer treatments [39]. In this sense, adapting the exercise program considering these issues and opting for a multidisciplinary management, e.g., simultaneous care and nutritional counseling, may be fundamental. Moreover, the fear of injury has emerged as another non-treatment-related factor potentially hindering adherence to an exercise program [39]. In this review, only three trials have investigated the safety of the exercise intervention. Although no adverse events directly attributable to exercise have been reported in these three studies, it is hard to state the safety of exercise, since the small sample size of the trials. Generally, exercise-related adverse events are rare but can be significant if they occur. In this sense, large studies with detailed adverse events reporting are needed to conclude safety with any certainty.

Studies investigating exercise during oncological treatments reported mixed results on head and neck cancer outcomes. Focusing on the type of exercise training, it seems that resistance training alone can not produce significant improvements in lean body mass, cardiorespiratory fitness, strength, fatigue, and quality of life [32, 33, 37]. Instead, combined aerobic and resistance training appears more beneficial and capable of increasing the outcomes mentioned above [28–31]. Whereas for the enhancements of some outcomes, such as cardiorespiratory fitness, aerobic training is fundamental, for others, the superiority of the combined aerobic and strength intervention with respect to the resistance training alone may seem unusual. This is the case, for instance, of muscle mass. Indeed, the gain of muscle mass is highly stimulated by resistance training but surprisingly, the studies that investigated resistance training as an intervention did not find improvements, while the combination of aerobic and resistance has been shown to promote protein synthesis. A possible explanation could be related to the control of inflammatory levels. Inflammation is associated with weight loss in patients with head and neck cancer [40], and it is a recognized hallmark of cancer cachexia [41]. Aerobic exercise may have an anti-inflammatory role, able to counteract catabolism and synergistically enhance the anabolic effect of strength training [42]. Since patients affected by head and neck cancer are at high risk of developing cachexia and frequently experience a severe depletion in skeletal muscle mass during treatments, impacting survival, quality of life, and treatment tolerance [43], finding approaches, such as full body physical exercise, to offset the usual deterioration is fundamental.

Finally, regarding the post-treatment phase, just one study has been published [34]. In this context, progressive resistance training improved lean body mass, strength, and quality of life. Nevertheless, it is necessary to increase the number of investigations as well as the proposed interventions and the outcomes evaluated to understand the real potential of exercise during the recovery phase of head and neck cancer.

The present review has limitations that should be noted. Only a limited number of studies with small sample sizes, to date, have explored the effect of exercise in patients with head and neck cancer during anticancer treatments. This may suggest to cautiously interpret the results since most studies were not appropriately powered to detect possible clinically relevant differences in all outcomes. Investigations testing the effect of full-body physical exercise as prehabilitation or during survivorship are practically absent. Moreover, heterogeneity in included disease subtypes and assessment tools emerged across the investigations, making the results difficult to compare and generalize.

## Conclusion

In summary, full-body physical exercise, mainly if composed of aerobic and resistance components, may help to mitigate some side effects commonly experienced by patients with head and neck cancers during anticancer therapies. Moreover, the present systematic review highlights the necessity to implement future studies with a solid design to investigate those underexplored areas, such as the prehabilitation one, and fully understand the role of exercise in supporting patients with head and neck cancer.

### Supplementary information

Below is the link to the electronic supplementary material.

Supplementary file1 (DOCX 135 KB)

## Data Availability

The data are available upon request from the authors.

## Code availability

N/A.
