# Supplementary material for: Effect of exercise across the head and neck cancer continuum: a systematic review of randomized controlled trials
Source: Support Care Cancer. 2023 Nov 4;31(12):670. doi: 10.1007/s00520-023-08126-2 (PMC10625510; doi:10.1007/s00520-023-08126-2)
Supplement: Supplementary file 1 — Supplementary file1 (DOCX 135 KB) [file 520_2023_8126_MOESM1_ESM.docx]

**Supplementary materials**

**Title:** Explore the effect of exercise across the head and neck cancer continuum: a systematic review of randomized controlled trials

- 1. Search strategy
  2. Full-text-assessed and reason for their exclusion
  3. Risk of bias assessment
  4. Tools for outcome assessment

**1.1 Search strategy**

Comprehensive search on:

1. PubMed/Medline
2. Cochrane Central Register of Controlled Trials (CENTRAL)
3. EBSCO Sports Medicine Database (SPORTDiscus)

The research was conducted combining relevant keywords. In particular three key-components have driven the research:

1. *Physical exercise*

“Physical activity” [Title/Abstract] OR “Muscle contraction” [Title/Abstract] OR “Exercise” [Title/Abstract] OR “Physical exercise” [Title/Abstract] OR “Exercise therapy” [Title/Abstract] OR “Resistance training” [Title/Abstract] OR “Walking” [Title/Abstract] OR “Circuit-based training” [Title/Abstract] OR “Strength training” [Title/Abstract] OR “Weight-bearing exercise” [Title/Abstract] OR “Aerobic training” [Title/Abstract] OR “Cardiorespiratory training” [Title/Abstract] OR “Fitness” [Title/Abstract] OR “Endurance training” [Title/Abstract] OR “Exercise prescription” [Title/Abstract] OR “Muscle strength” [Title/Abstract]

1. *Cancer*

“Neoplasm” [Title/Abstract] OR “Cancer” [Title/Abstract] OR “Tumor” [Title/Abstract] OR “Malignancy” [Title/Abstract]

1. *Head & neck*

“Head” [Title/Abstract] OR “Neck” [Title/Abstract] OR “Head and neck” [Title/Abstract] OR “Hypopharynx” [Title/Abstract] OR “Oral” [Title/Abstract] OR “Larynx” [Title/Abstract] OR “Oropharynx” [Title/Abstract] OR “Oral cavity” [Title/Abstract] OR “Lip” [Title/Abstract] OR “Oar” [Title/Abstract] OR “Tonsil” [Title/Abstract] OR “Nasopharynx” [Title/Abstract] OR “Nasal cavity” [Title/Abstract] OR “Paranasal sinus” [Title/Abstract] OR “Paranasal for middle ear” [Title/Abstract]

**1.2 Full-text-assessed and reason for their exclusion**

| **Authors (year)** | **Title** | **Reason of exclusion** |
| --- | --- | --- |
| Lavigne et al. (2020) | Feasibility of eccentric overloading neuromuscular electrical stimulation to improve muscle strength and muscle mass after treatment for head and neck cancer | No non-exercise intervention as comparator |
| Kristensen et al. (2020) | Effects of a multidisciplinary residential nutritional rehabilitation program in head and neck cancer survivors – results from the NUTRI-HAB randomized controlled trial | No full-body exercise as intervention |
| Chang et al. (2019) | The effectiveness of a nurse-led exercise and health education informatics program on exercise capacity and quality of life among cancer survivors after esophagectomy: a randomized controlled trial | No head and neck cancer |
| Xu et al. (2015) | A walk-and-eat intervention improves outcomes for patients with esophageal cancer undergoing neoadjuvant chemoradiotherapy | No head and neck cancer |
| Su et al. (2017) | The effect of home-based program and outpatient physical therapy in patients with head and neck cancer: a randomized controlled trial | No non-exercise intervention as comparator |
| McNeely et al. (2008) | Effect of exercise on upper extremity pain and dysfunction in head and neck cancer survivors | No non-exercise intervention as comparator |
| Sandmael et al. (2017) | Feasibility and preliminary effects of resistance training and nutritional supplements during versus after radiotherapy in patients with head and neck cancer: a pilot randomized trial | No non-exercise intervention as comparator |
| O’Neill et al.  (2018) | The RESTORE randomized controlled trial: Impact of a multidisciplinary rehabilitative program on cardiorespiratory fitness in esophagogastric cancer survivorship | No head and neck cancer |
| Lonbro et al. (2013) | Lean body mass and muscle function in head and neck cancer patients and healthy individuals – results from the DAHANCA 25 study | No cancer population as comparator |
| Lonbro et al. (2013) | Feasibility and efficacy of progressive resistance training and dietary supplements in radiotherapy-treated head and neck cancer patients – the DAHANCA 25A study | No non-exercise intervention as comparator |
| McNeely et al. (2004) | A pilot study of randomized controlled trial to evaluate the effects of progressive resistance exercise training on shoulder dysfunction caused by spinal accessory neurapraxia/neurectomy in head and neck cancer survivors | No non-exercise intervention as comparator |

**1.3 Risk of bias assessment**


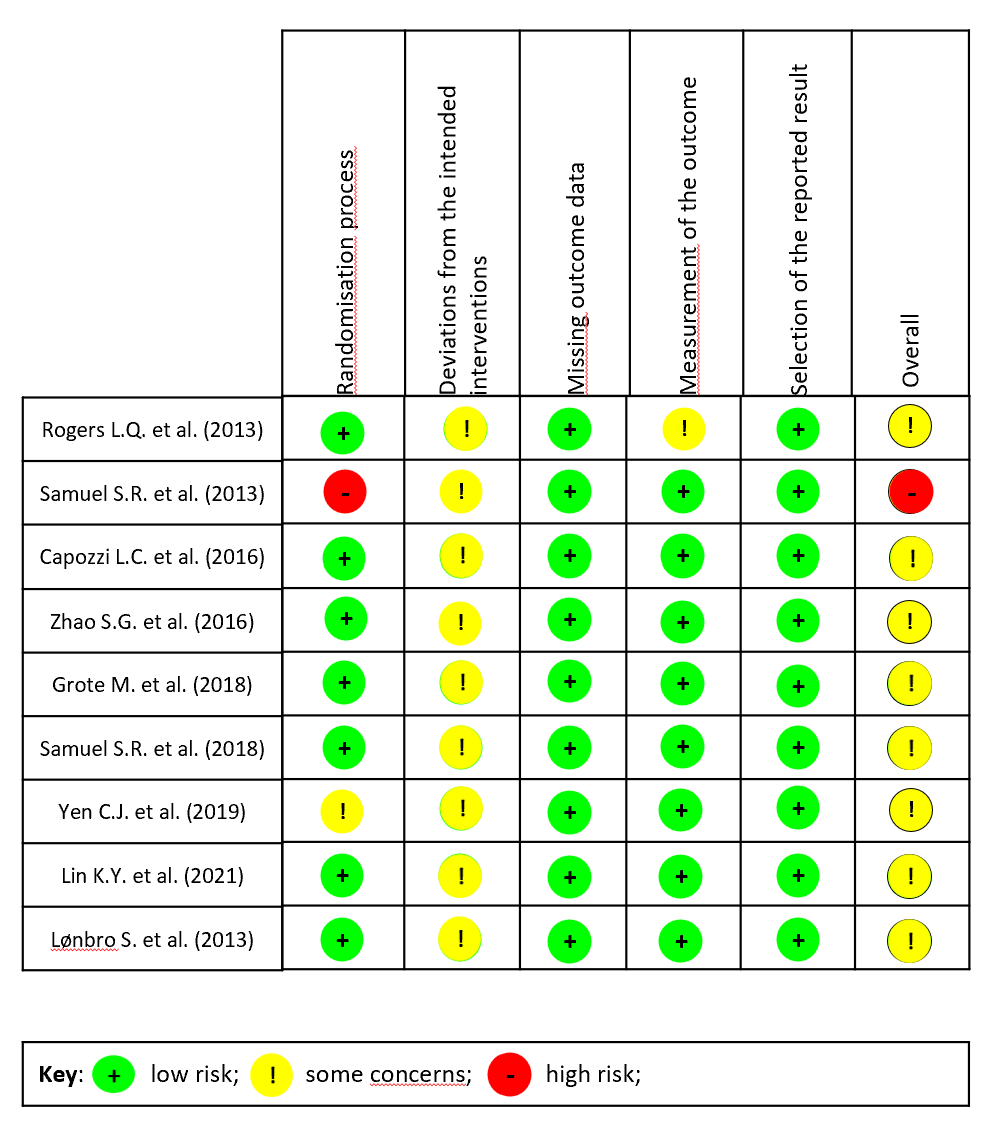


**1.4 Tools of outcomes assessment**

| **Study** | **Lean body mass** | **Fat mass** | **Cardiorespiratory fitness** | **Muscular strength** | **Balance** | **Flexibility** | **Physical activity** |
| --- | --- | --- | --- | --- | --- | --- | --- |
| Rogers L.Q. et al. (2013) | Bioimpedance analysis | NA | NA | Handgrip strength test; knee extensor; five chair rise and sit; | NA | NA | NA |
| Samuel S.R. et al. (2013) | NA | NA | Six minutes walking test |  | NA | NA | NA |
| Capozzi L.C. et al. (2016) | DEXA | DEXA | Six minutes walking test | Handgrip strength test; 30’’ chair stand; | NA | Sit and reach test | Godin Shepard Leisure Time Exercise questionnaire |
| Zhao S.G. et al. (2016) | DEXA | NA | Six minutes walking test | Handgrip strength test; elbow flexion and knee extension isokinetic dynamometer; | Time up and go test | NA | NA |
| Grote M. et al. (2018) | Bioimpedance analysis | Bioimpedance analysis | NA | NA | NA | NA | NA |
| Samuel S.R. et al. (2018) | NA | NA | Six minutes walking test | NA | NA | NA | NA |
| Yen C.J. et al.  (2019) | Bioimpedance analysis | Bioimpedance analysis | Six minutes walking test | NA | NA | NA | NA |
| Lin K.Y. et al. (2021) | Bioimpedance analysis | Bioimpedance analysis | Three minutes step test | 30’’ arm curl; 30’’ chair stand; | Time up and go test | Back scratch test; sit and reach test; |  |
| Lønbro S. et al. (2013) | DEXA | DEXA | NA | Isometric knee extensor/flexor; 30’’ chair stand; 30’’ arm curl; | 10 meters maximal gait speed | NA | NA |

| **Study** | **Blood pressure** | **Heart rate** | **Blood parameters** | **Treatment toxicities** | **Quality of life** | **Fatigue** | **Depression** | **Nutritional status** | **Sleep** |
| --- | --- | --- | --- | --- | --- | --- | --- | --- | --- |
| Rogers L.Q. et al. (2013) | NA | NA | NA | NA | FACT-H&N  FACT-G | NA | NA | NA | NA |
| Samuel S.R. et al. (2013) | NA | NA | NA | NA | Short form 36 | NA | NA | NA | NA |
| Capozzi L.C. et al. (2016) | NA | NA | NA | NA | FACT-Anemia  FHNSI-22 | NA | CES-D | PG-SGA | MOS sleep index |
| Zhao S.G. et al. (2016) | NA | NA | NA | NCI common toxicity criteria for adverse events | Short form 36  20-question head and neck quality of life questionnaire | NA | NA | NA | NA |
| Grote M. et al. (2018) | NA | NA | NA | NA | FAACT (Functional Assessment of Anorexia/cachexia Therapy) | Multidimensional Fatigue Inventory | NA | NA | NA |
| Samuel S.R. et al. (2018) | NA | NA | NS | NA | Short form 36 | NCCN (0-10) | NA | NA | NA |
| Yen C.J. et al.  (2019) | NS | NS | NA | NA | NA | NA | NA | NA | NA |
| Lin K.Y. et al. (2021) | NA | NA | NA | NA | EORTC QLQ C30  QLQ H&N35 | NA | NA | NA | NA |
| Lønbro S. et al. (2013) | NA | NA | NA | NA | EORTC QLQ C30 | NA | NA | NA | NA |
| Abbreviations: NA, not assessed, NS, not specified; | | | | | | | | | |
